# Supplementary material for: Discovery of a selective and biologically active low-molecular weight antagonist of human interleukin-1β
Source: Nat Commun. 2023 Sep 7;14:5497. doi: 10.1038/s41467-023-41190-0 (PMC10484922; doi:10.1038/s41467-023-41190-0)
Supplement: Supplementary file 1 — Supplementary Information [file 41467_2023_41190_MOESM1_ESM.pdf]

Supplementary Material to:
Discovery of a selective and biologically active low-molecular weight antagonist of human interleukin-1β

Ulrich Hommel[1,*], Konstanze Hurth[1,*], Jean-Michel Rondeau[1], Anna Vulpetti[1], Daniela Ostermeier[1], Andreas Boettcher[1], Jacob Peter Brady[2], Michael Hediger[1], Sylvie Lehmann[1], Elke Koch[1], Anke Blechschmidt[1], Rina Yamamoto[1], Valentina Tundo Dottorello[1], Sandra Haenni-Holzinger[1], Christian Kaiser[1], Philipp Lehr[1], Andreas Lingel[1], Luca Mureddu[3], Christian Schleberger[1], Jutta Blank[1], Paul Ramage[1], Felix Freuler[1], Joerg Eder[1], Frédéric Bornancin[1,*]

[1]Novartis Institutes for Biomedical Research
Novartis Campus
CH-4002 Basel

[2]Novartis Institutes for Biomedical Research
250, Massachusetts Avenue
Cambridge, MA 02139

[3]Leicester Institute of Structural and Chemical Biology
Department of Molecular and Cell Biology, University of Leicester
Leicester, LE1 7RH
United Kingdom

*Authors to whom correspondence should be addressed.
uli.hommel@icloud.com (ORCID 0000-0001-6532-9155)
konstanze.hurth@novartis.com (ORCID 0009-0000-9056-6101)
frederic.bornancin@novartis.com (ORCID 0000-0002-0152-6720)

Table of Contents

Supplementary Tables

Supp. Table 1: Small molecule screening data.

Supp. Table 2: X-ray data collection and refinement statistics for hIL-1β in complex with (*S*)-**2**.

Supp. Table 3: ^{15}N-CEST fitting parameters for hIL-1β and hIL-1β^{V47A} at 309 K.

Supp. Table 4: Chemical shifts for ^{15}N-CEST dips used in fitting hIL-1β and hIL-1β^{V47A}.

Supplementary Figures

Supp. Figure 1: Identification of compound **1** by ^{19}F NMR-based fragment screening and slow binding kinetics monitored by ^{1}H-NMR.

Supp. Figure 2: ^{13}C-NMR and ^{19}F-NMR reporter assays to monitor early structure activity relationships of analogues of **1**.

Supp. Figure 3: Determination of binding affinities for selected analogues of **1** by SPR.

Supp. Figure 4: Omit electron-density for compound (*S*)-**2** bound to hIL-1β

Supp. Figure 5: Binding site mapping of compound (*S*)-**2** to hIL-1β.

Supp. Figure 6: Temperature dependent and B1-field dependent ^{15}N-CEST profiles of residues involved in the conformationally excited state of hIL-1β.

Supp. Figure 7: ^{15}N-CEST profiles in the presence and absence of compound (*S*)-**2**.

Supp. Figure 8: Binding affinities of hIL-1β and hIL-1β^{V47A} to hIL-1R1 assessed by SPR.

Supp. Figure 9: Structural integrity of IL-1β^{V47A} monitored by NMR.

Supp. Figure 10: Comparison of ^{15}N-CEST profiles of hIL-1β and hIL-1β^{V47A}.

Supp. Figure 11: B1-field dependent ^{15}N-CEST profiles of residues of hIL-1β^{V47A}.

Supp. Figure 12: Direct observation of minor state peaks in hIL-1β^{V47A}.

Supp. Figure 13: Lineshape fitting of (*S*)-**1** binding to hIL-1β.

Supp. Figure 14: Comparison of hIL-36γ/A-552, hIL-1β/*(S)*-**2**, hIL-1α and the IL-1R1/ hIL-1β complex.

Supp. Figure 15: Compound (*S*)-**2** inhibits IL-1β-driven, but not IL-36γ-driven activity in a NanoBiT assay.

Supp. Figure 16: Synthesis scheme of compound (*S*)-**2**

Supplementary Methods

Synthesis

Reporter cell system for NanoBiT assay

Supplementary References

Supplementary Tables

Supplementary Table 1: Small molecule screening data.

**Category**	**Parameter**	**Description**
Assay	Type of assay	^{19}F-transverse relaxation NMR
	Target	Human interleukin-1β
	Primary measurement	Loss of ^{19}F-signal intensity
	Key reagents	n.a.
	Assay protocol	DOI: 10.1002/anie.202002463
Library	Library size	3,452 fragments
	Library composition	Mixtures of up to 32 fragments containing CF_3-, CF_2- and CF- groups (DOI: 10.1002/anie.202002463).
	Source	Proprietary and commercial compounds.
Screen	Format	Single tubes containing mixtures of up to 32 fragments.
	Concentration(s) tested	25 μM and 40 μM for CF_3 and CF-fragments, respectively, 4 μM for hIL-1β.
	Plate controls	NMR experiments are tube-based.
	Reagent/ compound dispensing system	TECAN
	Detection instrument and software	Bruker 600 MHz AVANCE III spectrometer; TOPSPIN
	Assay validation/QC	None
	Correction factors	None
	Normalization	None
Post-HTS analysis	Hit criteria	$I/I_0 < 0.7$
	Hit rate	Primary hit rate 0.8 %.
	Additional assay(s)	Hit validation by protein-observed ^{13}C-HMQC NMR
	Confirmation of hit purity and structure	^{1}H-NMR

Supplementary Table 2. X-ray data collection and refinement statistics for hIL-1β in complex with (*S*)-2. Related to Figure 4. Values in parentheses are for the highest resolution shell. Intensity statistics indicators and crystallographic R-factors are described in Evans, P.R. (2011)[1] and Brünger, A.T. (1992)[2], respectively. The definition of the $CC^{1/2}$ coefficient and its use are described in Karplus and Diederichs (2012, 2015)[3,4]. The free set represents a random selection of reflections (5%) not included in refinement. Values for the Ramachandran plot correspond to favored, allowed, and disallowed regions as defined by Molprobity[5].

**X-ray data collection**	
Wavelength (Å)	1.00004
Space group	$P2_1$
Cell dimensions : a, b, c (Å), β (°)	56.2, 60.1, 55.9, 114.8
Resolution (Å)	1.95 (1.98-1.95)
R_{merge}	0.117 (2.039)
R_{meas}	0.131 (2.256)
R_{pim}	0.058 (0.959)
I / σ(I)<br>$CC^{1/2}$	7.6 (0.9)<br>0.994 (0.326)
Completeness (%)	97.4 (97.0)
Multiplicity	5.1 (5.4)
**Crystallographic refinement**	
Resolution (Å)	50.74-1.95
No. reflections	24,237
R_{work} / R_{free}	0.208/ 0.241
No. atoms	
Protein<br>Ligand	2,424<br>2 x 35
Waters	202
B-factors (Å^2)	
Wilson B-factor<br>Protein (chain A ; B)<br>Ligand molecules (A201; B201)	39.4<br>45.8; 42.1<br>40.5; 28.6
Waters (chain W)	48.6
R.m.s. deviations	
Bond lengths (Å) / angles (°)	0.008 / 1.11
Ramachandran plot (%)	98.67, 1.33, 0.00

Supplementary Table 3: ^{15}N-CEST fitting parameters for hIL-1β and hIL-1β^{V47A} at 309 K. Excited state populations (p_b), exchange rates ($k_{ex} = k_1 + k_{-1}$) and reduced X^2 (X^2_{red}) values for IL-1β^{WT} and IL-1β^{V47A} mutant samples at 309 K. Errors were estimated by the program ChemEx (https://github.com/gbouvignies/ChemEx) using the standard deviations for fitted parameter values from 100 Monte Carlo simulations.

Sample	p_b (%)	k_{ex} (s^{-1})	X^2_{red}	Residues used for fit
hIL-1β	8.1 ± 0.8	22.3 ± 2.2	1.34	5, 47, 48, 83, 85, 86, 89, 90, 92, 94
hIL-1β^{V47A}	22.5 ± 1.8	10.8 ± 0.87	1.98	5, 47, 83, 85, 86, 89, 90, 96

Supplementary Table 4: Chemical shifts for ^{15}N-CEST dips used in fitting hIL-1β and hIL-1β^{V47A}. Chemical shifts for major and minor ^{15}N-CEST dips for hIL-1β samples at 309 K (hIL-1β and hIL-1β^{V47A} are indicated by subscripts WT and V47A respectively). $\Delta\varpi_{WT}$ and $\Delta\varpi_{V47A}$ are the differences in ppm between the major and minor CEST dips for WT and V47A respectively.

Residue	$Major_{WT}$ (ppm)	$Minor_{WT}$ (ppm)	$\Delta\varpi_{WT}$ (ppm)	$Major_{V47A}$ (ppm)	$Minor_{V47A}$ (ppm)	$\Delta\varpi_{V47A}$ (ppm)
S5	118.468	116.621	-1.847	117.114	116.075	-1.039
V47/A47	118.486	120.467	1.981	128.806	126.013	-2.793
Q48	120.402	122.148	1.746	-	-	-
E83	123.989	122.633	-1.356	124.217	122.824	-1.393
V85	117.255	123.676	6.421	117.484	123.829	6.345
D86	122.072	125.714	3.642	122.037	125.676	3.639
N89	114.618	116.746	2.128	114.495	116.447	1.952
Y90	116.481	120.561	4.080	116.728	120.317	3.589
K92	116.798	118.731	1.933	-	-	-
K94	118.134	120.021	1.887	-	-	-
E96	-	-	-	122.705	121.515	-1.190

Supplementary Figures

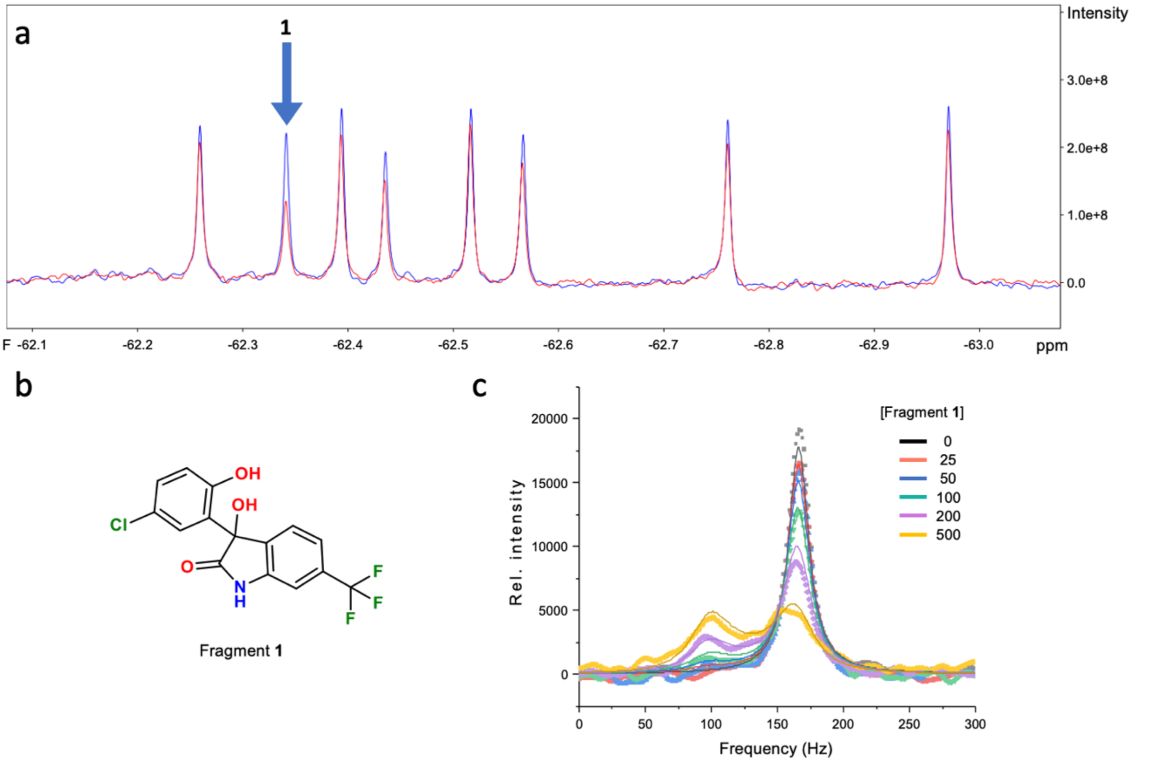

Supplementary Fig. 1. Identification of compound 1 by ^{19}F NMR-based fragment screening and slow binding kinetics monitored by ^{1}H-NMR. a ^{19}F-NMR spectrum of one fragment mixture screened for hIL-1β binders. The mixture design has been described earlier[6]. An excerpt of a ^{19}F-NMR spectrum covering the spectral range from -62.1 ppm to -63.1 ppm is shown. The signals of 8 fragments out of 32 included in this mixture are shown in the absence (blue) and presence (red) of the protein. Reduction of the signal representing fragment **1** at -62.35 ppm is highlighted, indicating an interaction with the protein. **b** Chemical structure of fragment **1**. **c** Non-linear least squares global fit of traces through a peak corresponding to Leu60 $C^{\delta 1}H_3$ in ^{1}H-^{13}C-HMQC spectra to the equation describing a two-site exchange process[7]. The protein concentration was 50 μM in PBS, pH 7.4 at 296 K. A global fit of all six spectra yielded K_D = 520 μM, k_{on} = 1.2 x 10^5 $M^{-1}s^{-1}$, k_{off} = 63 s^{-1}.

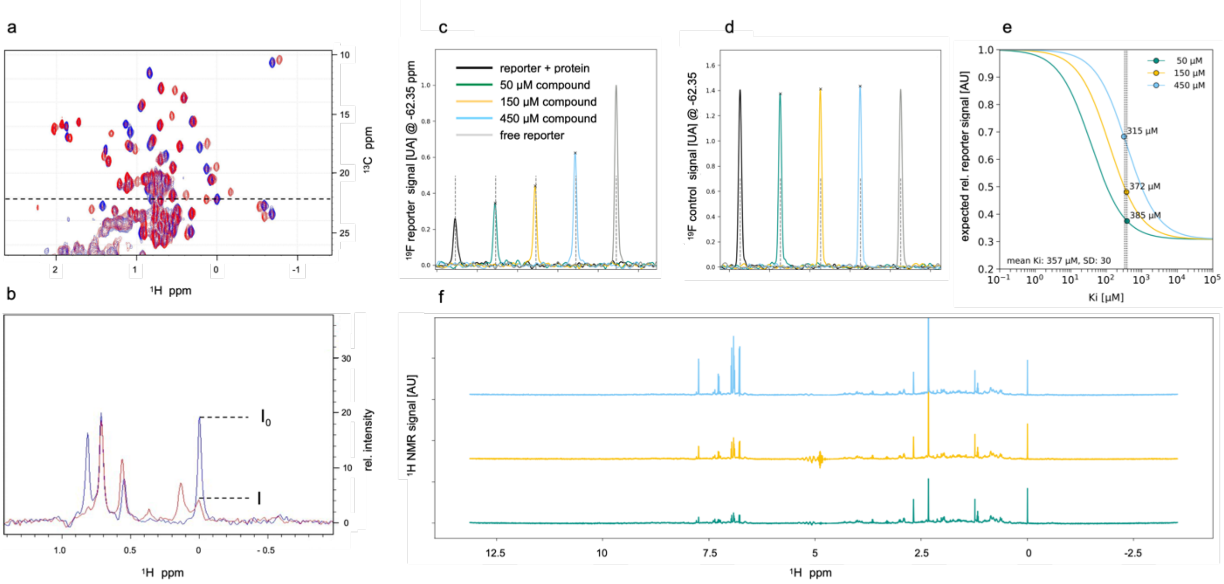

Supplementary Figure 2. ^{13}C-NMR and ^{19}F-NMR reporter assays to monitor early structure activity relationships of analogues of 1. a, b Early structure activity relationships monitored by protein-observed and **c-f** ^{19}F-NMR reporter assays, exemplified for compound **8**. **a** ^{1}H-^{13}C-HMQC spectra of hIL-1β (50 μM) were acquired in the presence (red) and absence (blue) of 500 μM compound and rows were extracted at the ω_1-frequency of the peak corresponding to Leu60 $C^{\delta 1}H_3$. **b** The degree of protein binding was estimated from the ratio I/I_0, where I and I_0 correspond to the signal in the presence and absence of compound, respectively. **c** For the assessment of the K_I-values, the signal intensity of a reporter molecule (*S*)-**1** was monitored in ^{19}F-transverse relaxation experiments run in the absence and presence of increasing amounts of compound. **d** All intensities were scaled to the intensity of an internal standard (all signals in panel **c** and **d** are shifted on the X-axis for clarity). **e** K_I-values were retrieved from theoretical binding curves calculated for each compound concentration[8]. **f** One-dimensional ^{1}H-NMR spectra were recorded for each assay mixture to assess the structural integrity and solubility of the compound in the sample.

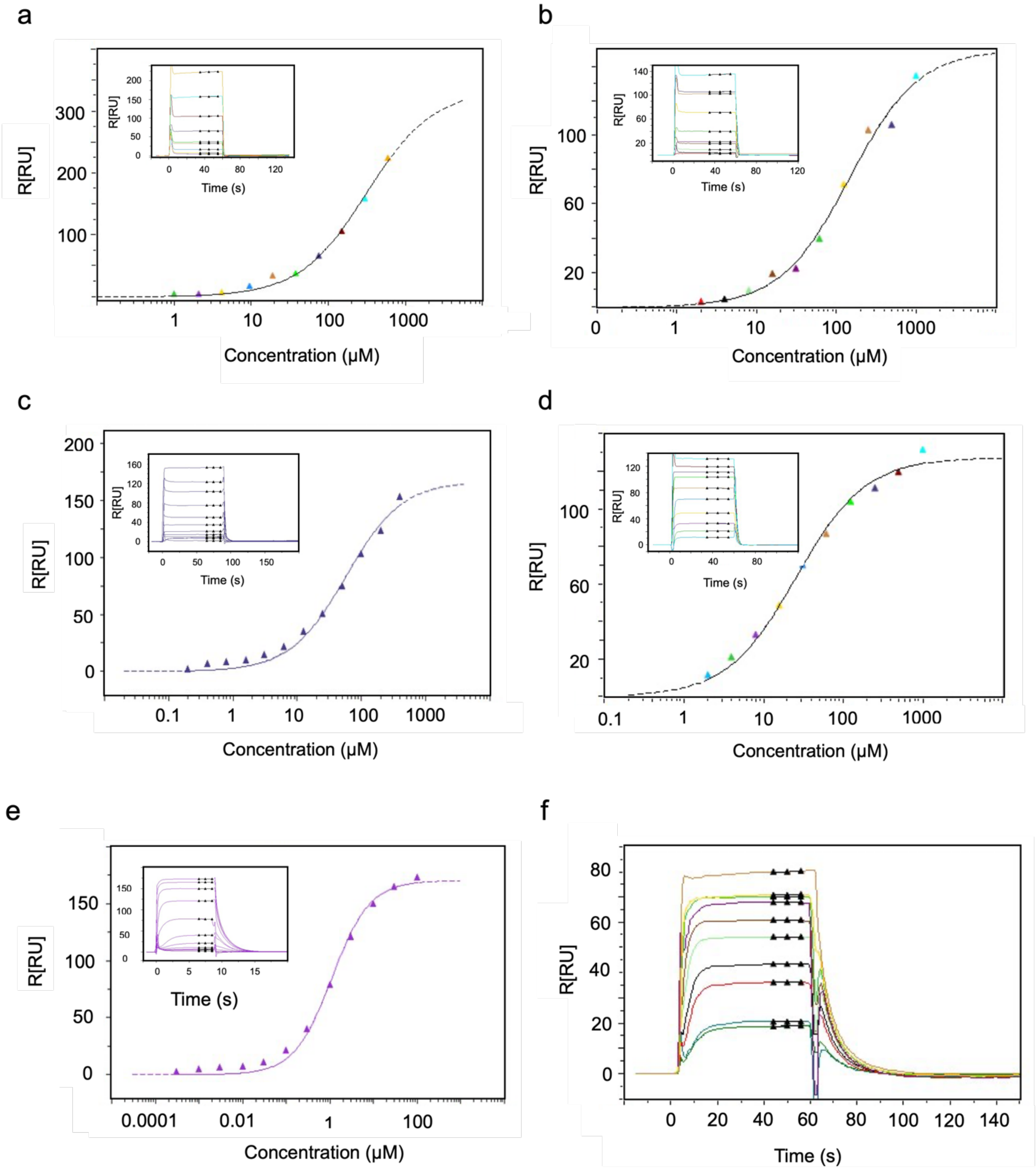

Supplementary Figure 3. Determination of binding affinities for selected analogues of 1 by SPR. **a - d** Graphs of exemplary experiments are given, where the saturation of hIL-1β immobilized on a chip was monitored at various concentrations of compound **3, 12, 10**, and **11,** respectively. The saturation reached at equilibrium (insert) was plotted versus the administered concentration and the equilibrium binding constant was derived from a non-linear least square fitting routine. **e** equilibrium and **f** kinetic binding of (*S*)-**2** to IL-1β (k_{on} = 1.15 x 10^5 M^{-1} s^{-1} and k_{off} = 0.12 s^{-1}).

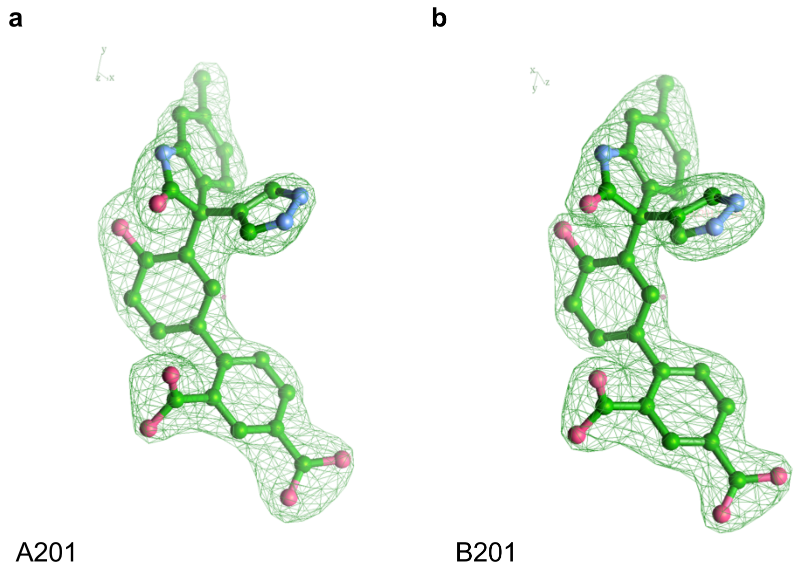

Supplementary Figure 4. Omit electron-density for compound (*S*)-2 bound to hIL-1β. **a** Omit electron-density (Polder map[9]) for compound (*S*)-**2** bound to IL-1β chain A, contoured at 4.5σ (green mesh). **b** Omit electron-density (Polder map[9]) for compound (***S***)-2 bound to IL-1β chain A, contoured at 4.5σ (green mesh)

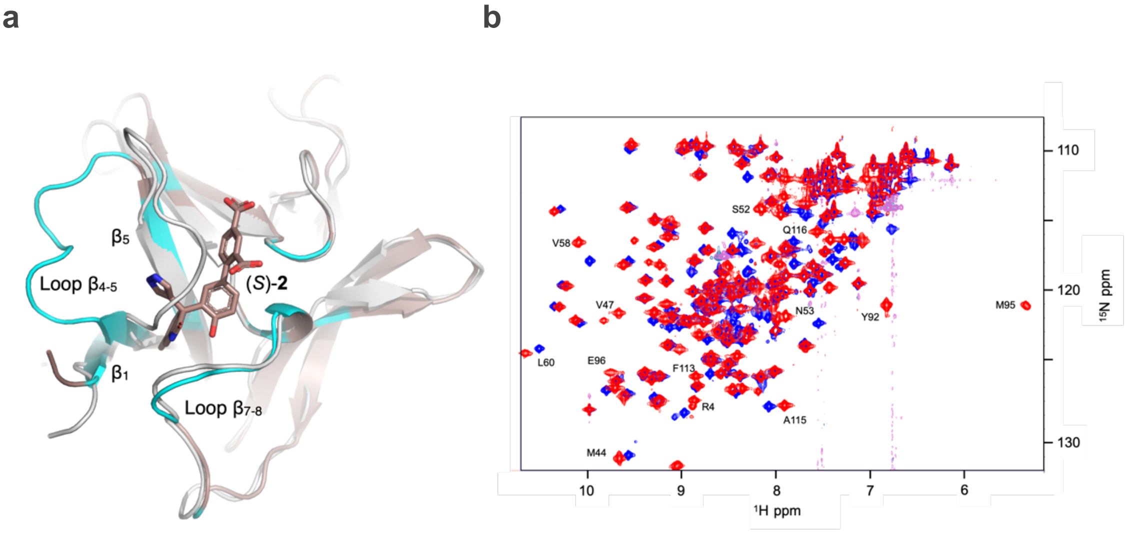

Supplementary Figure 5. Binding site mapping of fragment (*S*)-2. **a** Chemical shift changes (cyan) induced upon binding of (*S*)-**2** to hIL-1β were calculated in AnalysisAssign[10] for backbone amide hydrogen and nitrogen atoms and mapped onto the X-ray structure of the hIL-1β/(*S*)-**2** complex (brown), which is superimposed onto the X-ray structure of hIL-1β as observed in its ternary complex with IL-1R1 and IL-1RAcP (grey, PDB: 4DEP[11]). **b** Overlay of ^{1}H-^{15}N-HMQC spectrum of hIL-1β in the presence (red) and absence (blue) of (*S*)-**2.** Chemical shifts have been deposited (BMRB 51938).

a

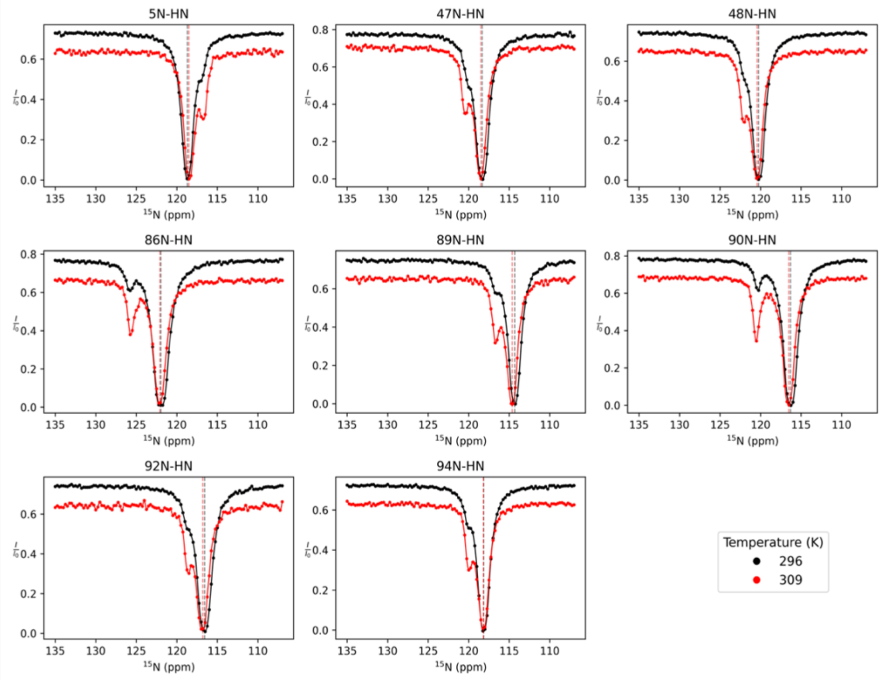

b

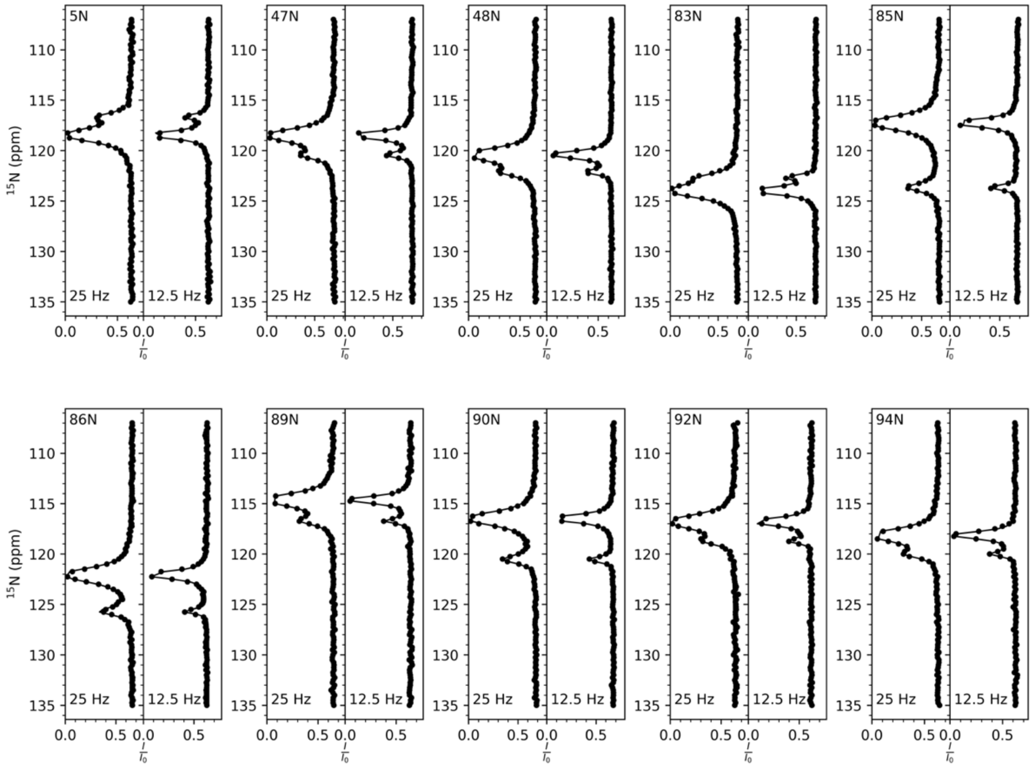

Supplementary Figure 6. Temperature dependent and B1-field dependent ^{15}N-CEST profiles of residues involved in the conformationally excited state of hIL-1β. a ^{15}N-CEST traces are shown for selected residues of hIL-1β at 296 K (black lines) and 309 K (red lines). **b** ^{15}N-CEST traces are shown for selected residues hIL-1β recorded at a B1 radiofrequency field strengths of 25 Hz and 12.5 Hz. Lines represent global non-linear least square fits of the data using the programme ChemEx (https://github.com/gbouvignies/ChemEx).

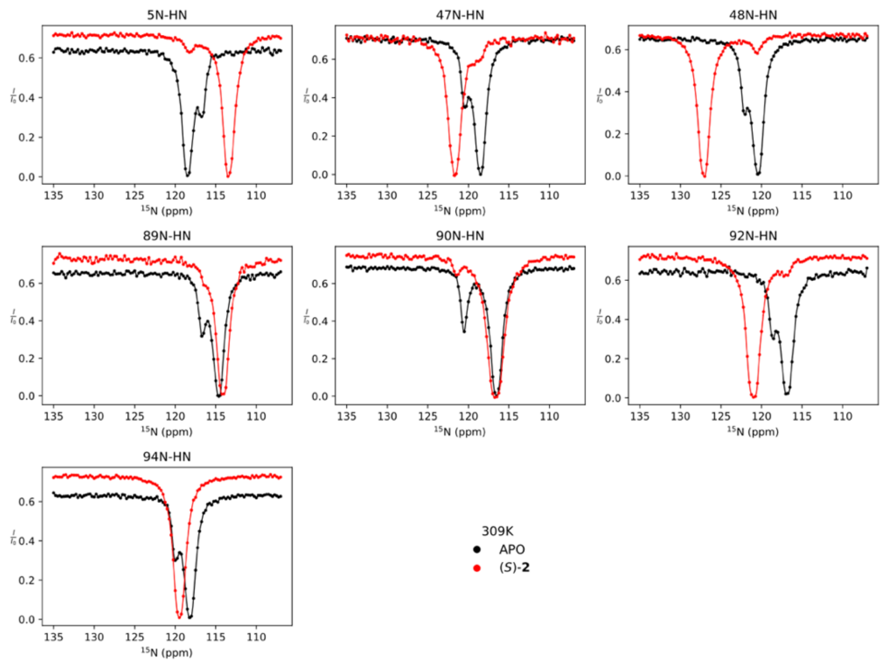

Supplementary Figure 7. **^{15}N-CEST profiles in the presence and absence of compound (*S*)-2.** ^{15}N-CEST traces are shown for selected residues in the presence (red) and absence (black) of compound (*S*)-**2**.

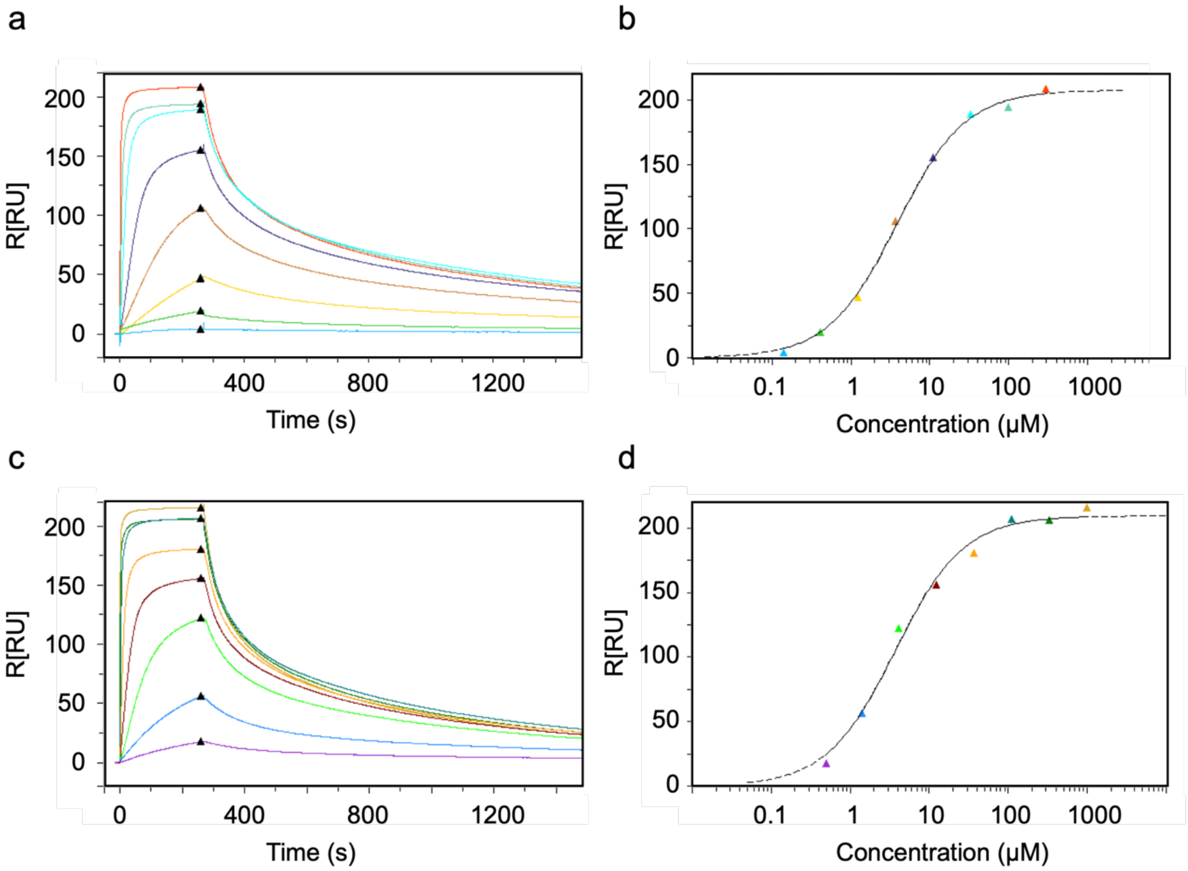

Supplementary Figure 8. Binding affinities of hIL-1β and hIL-1β^{V47A} to hIL-1R1 assessed by SPR. Binding of hIL-1β (**a**, **b**) and hIL-1β^{V47A} (**c**, **d**) to hIL-1R1 monitored by SPR. The derived affinity constants are K_D = 3.2 ±1.1 nM (N=2) and K_D = 3.9 ± 0.5 nM (N=3) for hIL-1β and hIL-1β^{V47A}, respectively. Source data are provided as a Source Data file.

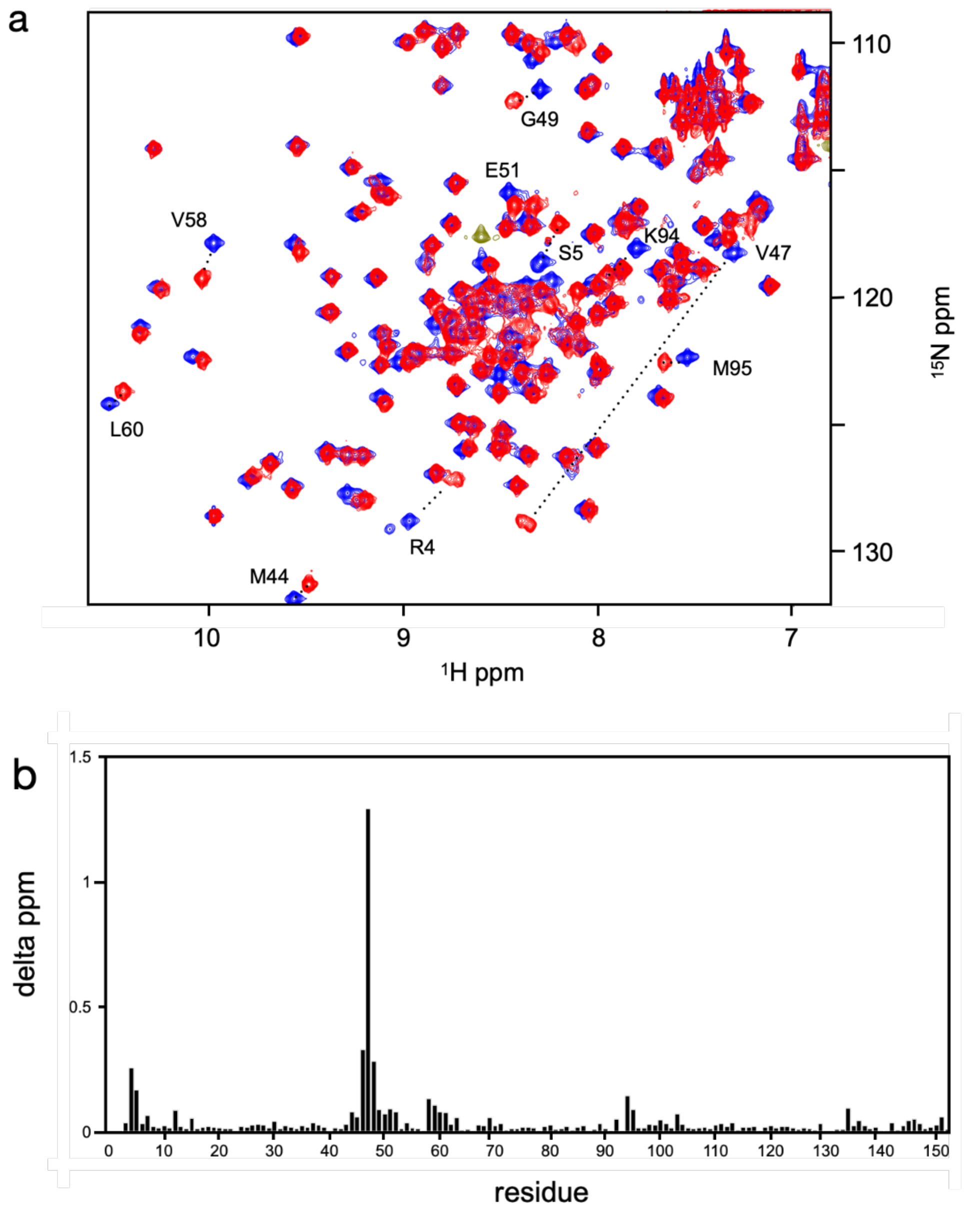

Supplementary Figure 9. Structural integrity of IL-1β^{V47A} monitored by NMR.

Characterization of hIL-1β^{V47A} by NMR. **a** Overlay of ^{1}H-^{15}N-HMQC spectra for hIL-1β (blue) and hIL-1β^{V47A} (red). **b** Weighted chemical shift differences were calculated in AnalysisAssign[10] for backbone amide hydrogen and nitrogen atoms and plotted versus the amino acid sequence of hIL-1β.

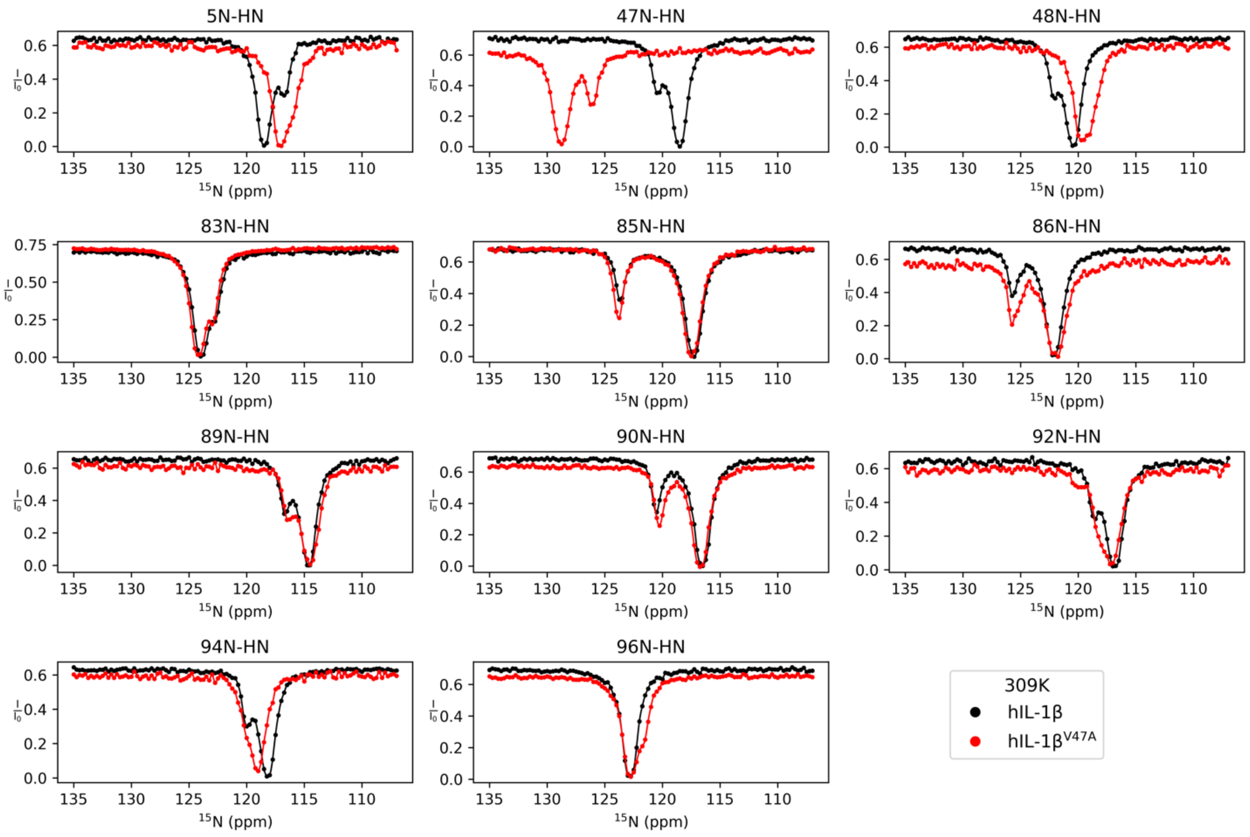

Supplementary Figure 10. Comparison of ^{15}N-CEST profiles of hIL-1β and hIL-1β^{V47A}.

^{15}N-CEST traces are overlayed for selected residues from hIL-1β (black) and hIL-1β^{V47A} (red) at 309 K.

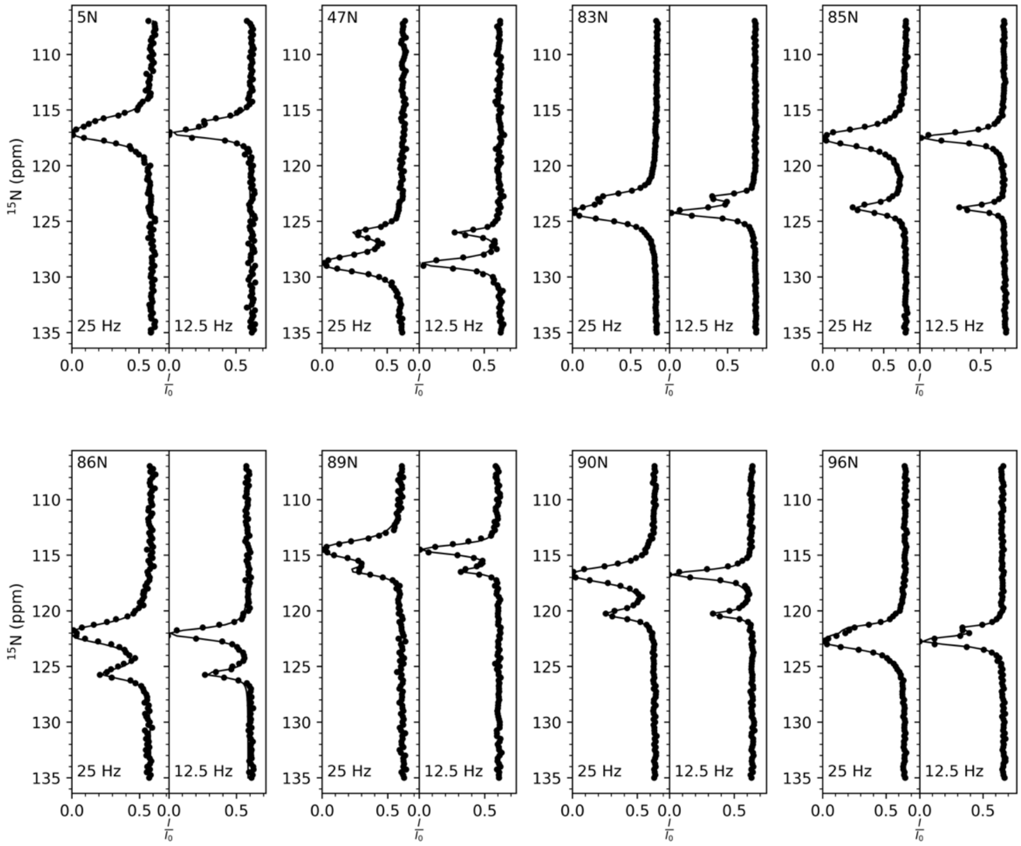

Supplementary Figure 11: B1-field dependent ^{15}N-CEST profiles of residues of hIL-1β^{V47A}. ^{15}N-CEST traces are shown for selected residues of hIL-1β^{V47A} recorded at a B1 radiofrequency field strengths of 25 Hz and 12.5 Hz. Lines represent global non-linear least square fits of the data using the programme ChemEx (https://github.com/gbouvignies/ChemEx).

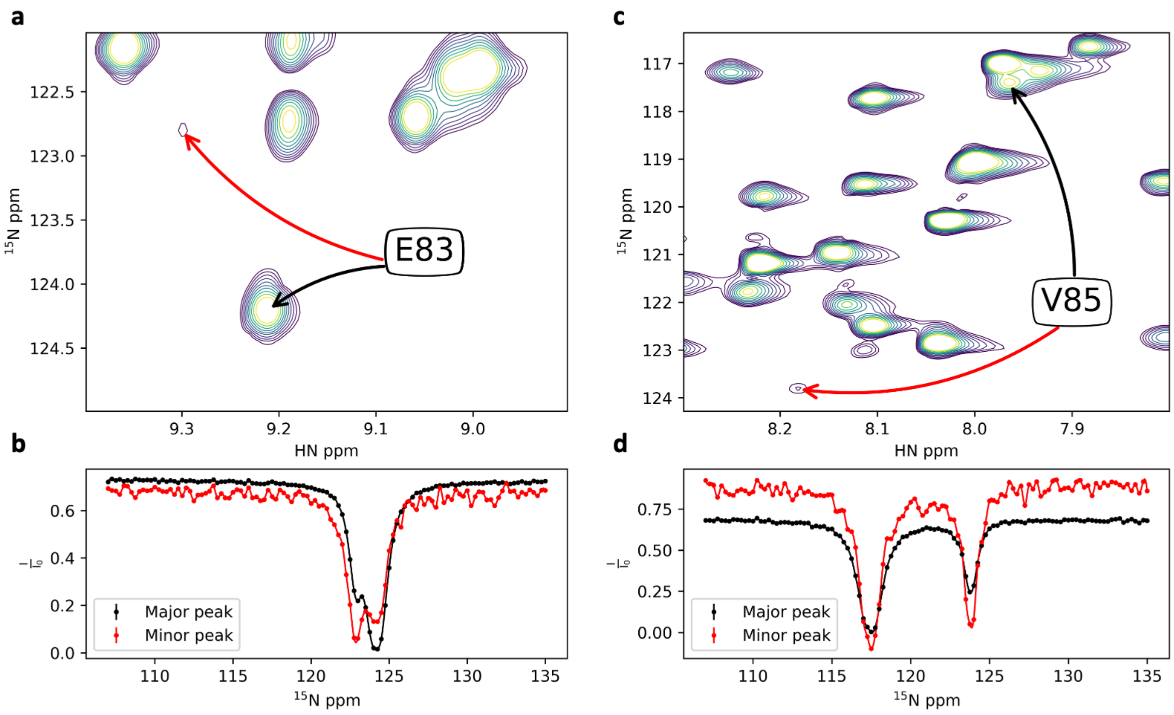

Supplementary Figure 12: Direct observation of minor state peaks in hIL-1β^{V47A}. **a** ^{15}N ALSOFAST-HMQC spectrum of hIL-1β^{V47A} at 309 K highlighting minor (red arrow) and major (black arrow) peaks for E83. **b** ^{15}N-CEST profiles for minor (red) and major (black) peaks for E83. **c** ^{15}N ALSOFAST-HMQC spectrum of hIL-1β^{V47A} at 309 K highlighting minor (red arrow) and major (black arrow) peaks for E83. **d** ^{15}N-CEST profiles for minor (red) and major (black) peaks for V85.

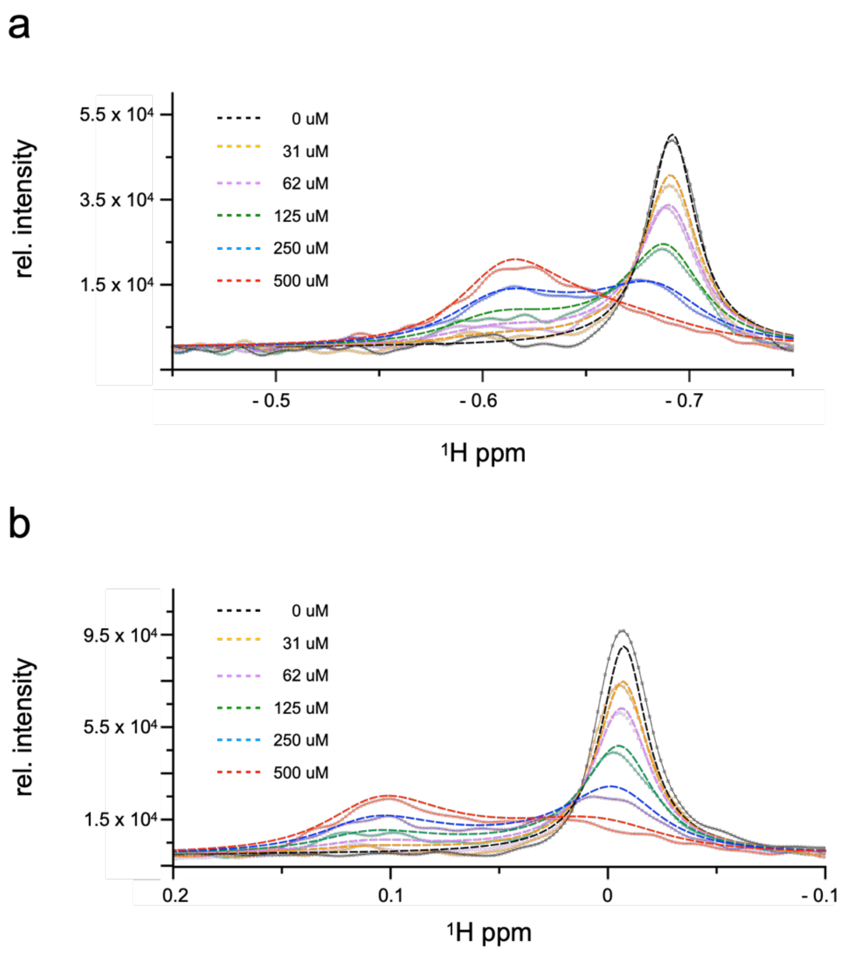

Supplementary Figure 13. Lineshape fitting of (*S*)-1 binding to hIL-1β. Lineshape fitting of **a** the $C^{\delta1}H3$- and **b** the $C^{\delta2}H3$-resonances of Leu60, respectively, upon titration of hIL-1β with increasing amounts of (*S*)-**1**. Broken lines correspond to the global best fit to the experimental data (K_D = 324 ± 5 μM, k_{off} = 79 ± 2 s^{-1} and K_D = 250 ± 36 μM, k_{off} = 76 ± 2 s^{-1}, for **a** and **b**, respectively).

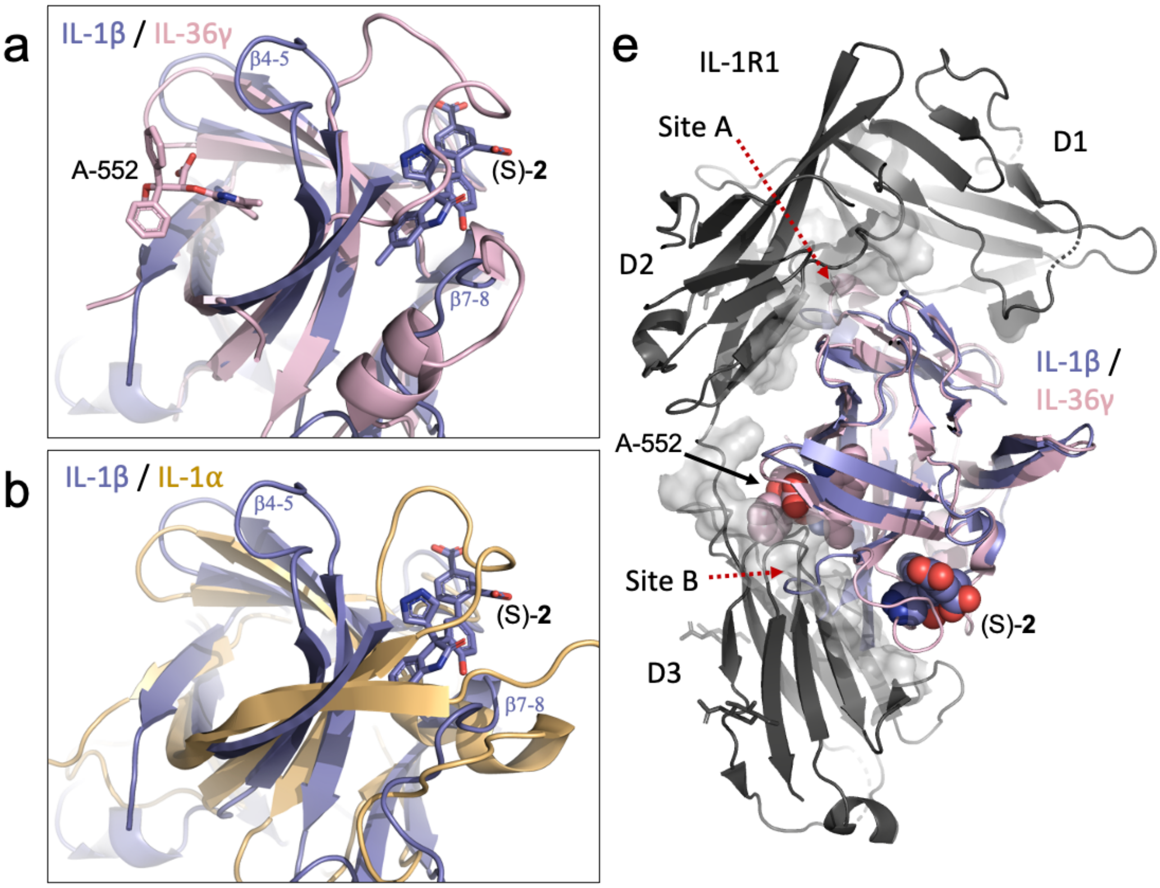

c

Cytokine		Q-score	Rmsd (Å)	Sequence Identity (%)	Nb of aligned residues
IL-1β	(2I1B.PDB)	1.0000	0.00	100.0	153/153
IL-1α	(2KKI.PDB)	0.4939	2.03	27.1	129/151
IL-36γ	(6P9E.PDB)	0.6177	1.60	23.9	134/148

d

Cytokine		3	45	46	47	50	57	58	59	91	92			93			94	95			96	97	100	102	115
IL-1β	(2I1B.PDB)	V	S	F	V	E	P	V	A	P	K	-	-	K	-	-	K	M	-	-	E	K	V	N	A
IL-1α	(2KKI.PDB)	m	G	A	y	s	T	V	I	I	p	-	-	K	t	-	I	T	g	s	E	T	L	F	V
IL-36γ	(6P9E.PDB)	c	I	T	c	p	P	I	Y	M	d	I	y	G	q	p	E	P	-	-	V	K	L	Y	V

Supplementary Figure 14. Comparison of hIL-36γ/A-552, hIL-1β/*(S)*-2, hIL-1α and the IL-1R1/ hIL-1β complex. **a** Superposition of the IL-36γ/A-552 complex (6P9E[12], light pink) onto the hIL-1β/(S)-2 complex (this work, slate); the two compounds are shown in stick representation. Note the different location of the two binding sites. **b** Superposition of unliganded IL-1α (2KKI[13], gold) onto the hIL-1β/(S)-2 complex (this work, slate); note the poor match of these two IL-1 cytokines around the (S)-**2** binding site. **c** Statistics produced by the superposition program GESAMT[14] from the CCP4 program suite[15] for pairwise comparisons between IL-1β[16], IL-1α[13] and IL-36γ[12]. **d** Conservations of amino-acid residues lining the (S)-**2** binding site in IL-1α[13] and IL-36γ[12]. The alignment was produced by the program GESAMT[14] from CCP4[15]. Amino-acid in uppercase occupy structurally-equivalent

positions in the respective three-dimensional structures while those shown in lowercase do not. Non-conserved residues are highlighted in red. **e** Superposition of the IL-36γ/A-552 (6P9E[12], light pink) and hIL-1β/IL-1R1 (4DEP[11], dark grey) complexes onto the hIL-1β/*(S)*-**2** complex (this work, slate). Note that both A-552 and (S)-2, while binding in topologically distinct binding sites, are facing the third Ig-domain (D3) of IL-1R1 (Site B of the cytokine/receptor interface, shown as a grey transparent surface); this overlay suggests a similar mode of action for these two compounds, provided IL-36γ binds to its cognate receptor IL-36R in a similar way as IL-1β to IL-1R1.

Compound (*S*)-2	Experiment #1		Experiment #2		Experiment #3	
IC_{50} (µM)<br>NanoBiT<br>**IL-1β**	22	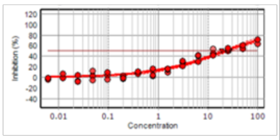	16	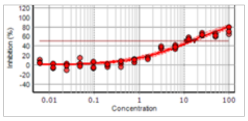	27	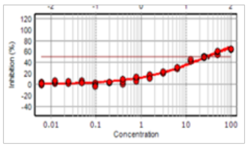
IC_{50} (µM)<br>NanoBiT<br>**IL-36γ**	-	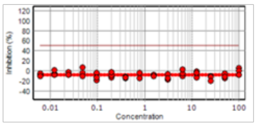	-	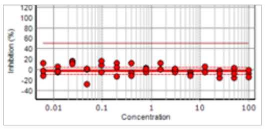	-	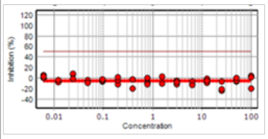

Supplementary Fig. 15. Compound (*S*)-2 inhibits IL-1β-driven, but not IL-36γ-driven activity in a NanoBiT assay. Comparative NanoBiT assays with IL-1β or IL-36γ, evaluating the effect of a concentration range of compound (*S*)-**2**. The compound inhibited the reporter activity driven by IL-1β but was without effect on IL-36γ-driven activity. Each experiment features triplicate determinations over a concentration range of compound.

Supplementary Methods

Synthesis

General information.

All reagents were purchased from commercial sources and used without further purification. ^{1}H and ^{13}C NMR were measured on various Bruker Avance spectrometers at room temperature and data are reported as follows: chemical shift (p.p.m., units) from an internal standard, multiplicity (s = singlet, d = doublet, dd = double doublet, t = triplet, q = quartet, m = multiplet, and br = broad), coupling constant *J* (Hz), and integration. High Resolution Mass Spectrometry analyses were performed by using electrospray ionization in negative ion modus (Ultimate 3000 from Dionex). The elemental composition was derived from the mass spectra acquired at the high resolution of about 70,000 on an Q Exactive Plus mass spectrometer (Thermo Scientific). The high mass accuracy below 2 ppm was obtained by using a lock mass.

LC-MS method: Instrument: Shimadzu LC-20AB Series LC-MS system with Shimadzu LC-MS-2020 mass spectrometer. Column: Kinetex EVO C18 30*2.1mm, 5 μm at 50°C; detector: PDA (220 nm and 254 nm); mobile phase: eluent A: 0.0375% TFA in H_2O, v/v, eluent B: 0.01875% TFA in ACN, v/v; gradient: 5% to 95% B in 1.2 min; flow 1.5 mL/min.

Analytical chiral SFC method: Instrument: Shimadzu LC-30-ADSF; column: Cellucoat 50×4.6mm I.D., 3 μm; detector: UV (220 nM); mobile phase: eluent A: sc-CO_2, eluent B MeOH (0.05% DEA); gradient: 5% to 40% B in 1.8 min; flow: 3 mL/min; column temperature: 35°C; pressure: 100 bar.

Preparative chiral SFC method: Instrument: Waters 80Q; column: Daicel Chiralpak OD column, 250×25mm I.D., 10 μm particle size; detector: UV (220 nM); mobile phase: eluent A: sc-CO_2, eluent B: MeOH (0.1% NH_4OH); isocratic elution: 40% B; flow: 70 mL/min; column temperature: 35°C; pressure: 100 bar.

Preparative HPLC method: Instrument: Gilson 281; column: Phenomenex Synergi C18 150*25mm*10 μm; detector PDA (220 nM and 254 nM); mobile phase: eluent A: water (0.225% formic acid v/v), eluent B: ACN; gradient: 22 to 52% B in 9.0 min; flow 25 mL/min; column temperature: room temperature.

Abbreviations: ACN: acetonitrile; aq.: aqueous; DEA: diethyl amine; EtOAc: ethyl acetate; FC: flash chromatography on silica gel; MeOH: methanol; N_2: nitrogen; PE: petroleum ether; RM: reaction mixture, RT: room temperature; Rt: retention time; HPLC: high performance liquid chromatography; org.: organic; Pd(dppf)Cl_2: 1,1′-Bis-(diphenylphosphino)-ferrocen]-dichloropalladium(II); sat.: saturated; soln.: solution; SEM: 2-(trimethylsilyl)ethoxymethyl; SFC: supercritical fluid chromatography; TFA: trifluoroacetic acid.

Compound 1, (*R*,*S*)-3-(5-chloro-2-hydroxyphenyl)-3-hydroxy-6-(trifluoromethyl)indolin-2-one (CAS 183720-17-4) was identified as a hit from the LEF4000 library and has been described before as well as its enantiomers, compound (*S*)-**1,** (*S*)-3-(5-chloro-2-hydroxyphenyl)-3-hydroxy-6-(trifluoromethyl)indolin-2-one) (CAS 183720-26-5) and compound (*R*)-**1**, (*R*)-3-(5-chloro-2-hydroxyphenyl)-3-hydroxy-6-(trifluoromethyl)indolin-2-one) (CAS 183720-27-6).

Compound (*S*)-2 was prepared in 6 steps according to Supplementary Figure 16.

Supplementary Figure 16: Synthesis of (*S*)-2.

(*S*)-**2** was synthesized in 6 steps from 6-methylindoline-2,3-dione by sequential addition of an aryl turbo-Grignard reagent (obtained from 4-iodo-1-((2-(trimethylsilyl)ethoxy)methyl)-1H-pyrazole) and of 4-bromophenol, followed by SEM deprotection, chromatographic enantiomer separation, a Suzuki coupling and ester hydrolysis.

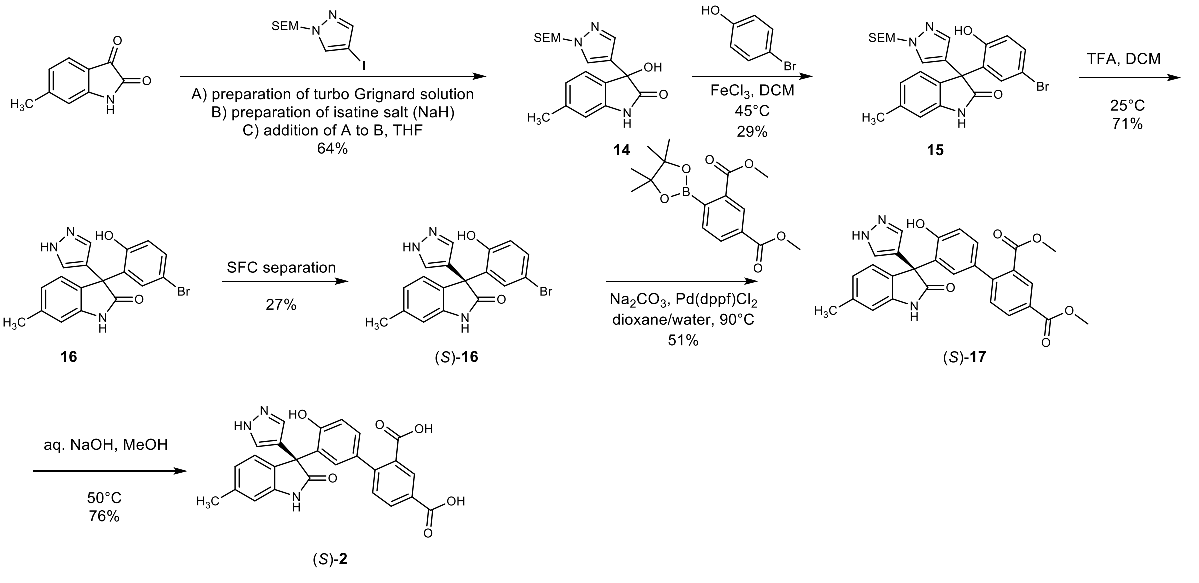

Compound 14: (*R*,*S*)-3-Hydroxy-6-methyl-3-(1-((2-(trimethylsilyl)ethoxy)methyl)-1*H*-pyrazol-4-yl)indolin-2-one:
To a soln. of 4-iodo-1-((2-(trimethylsilyl)ethoxy)methyl)-1*H*-pyrazole (CAS 220299-49-0; 80.5 g, 248.28 mmol) in THF (200 mL) was added i-PrMgCl •LiCl (191 mL, 1.3M in THF) at -20 °C and the RM was stirred for 0.5 h at -20 °C under N_2 to yield soln. A. At the same time, to a soln. of 6-methylindoline-2,3-dione (CAS 1128-47-8; 20.0 g, 124.10 mmol) in THF (200 mL) was added NaH (7.45 g, 186.25 mmol, 60% purity) at 0°C, then the mixture was stirred at 0°C for 0.5 h to afford soln. B, to which solution A was added at -20 °C. The RM was stirred at RT for 4.5 h, then poured into sat. aq. NH_4Cl soln. (2000 mL) and extracted with EtOAc (800 mL*3). The combined org. layers were washed with brine (200 mL), dried over Na_2SO_4, filtered and concentrated to give a residue that was purified by recrystallization from PE : EtOAc 5 : 1 (200 mL) to yield the title compound (31.0 g, 86.23 mmol, 64% yield) as a white solid. LC-MS: Rt=0.877 min, m/z=360.1 $[M+H]^+$; ^{1}H NMR (400 MHz, $CDCl_3$) δ = 8.32 (s, 1H), 7.61 (d, *J* = 0.6 Hz, 1H), 7.54 (s, 1H), 7.33 (d, *J* = 7.6 Hz, 1H), 6.92 (dd, *J* = 0.6, 7.6 Hz, 1H), 6.71 (s, 1H), 5.32 (d, *J* = 0.7 Hz, 2H), 3.97 (s, 1H), 3.63 - 3.44 (m, 2H), 2.35 (s, 3H), 0.93 - 0.79 (m, 2H), -0.03 - -0.06 (m, 9H).

Compound 15: (*R*,*S*)-3-(5-Bromo-2-hydroxyphenyl)-6-methyl-3-(1-((2-(trimethylsilyl)-ethoxy)methyl)-1*H*-pyrazol-4-yl)indolin-2-one:
A mixture of **Compound 14** (15.0 g, 41.72 mmol), 4-bromophenol (CAS 106-41-2; 14.44 g, 83.46 mmol) and $FeCl_3$ (3.38 g, 20.84 mmol) in DCM (450 mL) was stirred at 45°C for 3 h. The RM was diluted with water (1000 mL) and extracted with EtOAc (300 mL*3). The combined org. layers were dried over anhydrous Na_2SO_4, filtered and concentrated to give a residue that was purified by FC (PE : EA 0-60%) to yield the title compound (6.1 g, 12.63 mmol, 29% yield) as a yellow solid; LC-MS: Rt=1.047 min; m/z =536.1 $[M+Na]^+$; ^{1}H NMR (400 MHz, CD_3OD) δ = 7.57 (s, 1H), 7.54 (s, 1H), 7.22 (dd, *J* = 2.5, 8.5 Hz, 1H), 7.04 (d, *J* = 2.4 Hz, 1H), 6.93 - 6.88 (m, 1H), 6.85 - 6.80 (m, 1H), 6.76 (s, 1H), 6.63 (d, *J* = 8.5 Hz, 1H), 5.40 (s, 2H), 3.55 (t, *J* = 8.0 Hz, 2H), 2.33 (s, 3H), 0.90 - 0.81 (m, 2H), -0.03 - -0.06 (m, 9H).

Compound 16: (*R*,*S*)-3-(5-Bromo-2-hydroxyphenyl)-6-methyl-3-(1*H*-pyrazol-4-yl)indolin-2-one:

To a mixture of **Compound 15** (6.12 g, 11.89 mmol) in DCM (61.2 mL) was added TFA (30.6 mL) at 0°C. The RM was stirred at 25°C for 2 h, poured into aq. $NaHCO_3$ soln. (1000 mL) and extracted with EtOAc (300 mL*3). The combined org. layers were washed with brine (200 mL), dried over Na_2SO_4, filtered and concentrated to give a residue that was recrystallized from PE : EtOAc 2 : 1 (100 mL) to afford the title compound (3.25 g, 8.46 mmol, 71% yield) as a light yellow solid; LC-MS: Rt=0.848 min, m/z=384.0/386.0 $[M+H]^+$; ^1H NMR (400 MHz, CD_3OD) δ = 7.56 - 7.44 (m, 2H), 7.25 - 7.17 (m, 1H), 7.01 (d, *J* = 2.5 Hz, 1H), 6.93 - 6.88 (m, 1H), 6.85 - 6.80 (m, 1H), 6.76 (s, 1H), 6.62 (d, *J* = 8.4 Hz, 1H), 2.33 (s, 3H).

Compound (*S*)-16: (*S*)-3-(5-Bromo-2-hydroxyphenyl)-6-methyl-3-(1*H*-pyrazol-4-yl)indolin-2-one:
Compound **16** (1.8 g, 4.28 mmol) was separated by chiral SFC and concentrated to afford (*R*)-**16** and (*S*)-**16**, whose absolute configurations were assigned *post hoc* by correlation to the final products: Compound **(*R*)-16** (515 mg, 29% yield; white solid); LC-MS: Rt=0.839 min, m/z=384.0/386.0 $[M+H]^+$; ^1H NMR (400 MHz, CD_3OD) δ= 7.48 (s, 2H), 7.22 (dd, *J* = 2.5, 8.5 Hz, 1H), 7.01 (d, *J* = 2.5 Hz, 1H), 6.93 - 6.88 (m, 1H), 6.85 - 6.81 (m, 1H), 6.76 (s, 1H), 6.62 (d, *J* = 8.5 Hz, 1H), 2.34 (s, 3H); analytical chiral SFC: Rt=1.640 min, ee=94.1%. **Compound (*S*)-16** (485 mg, 27% yield; white solid); LC-MS: Rt=0.836 min, m/z=384.0/386.0 $[M+H]^+$; ^1H NMR (400 MHz, CD_3OD) δ = 7.48 (s, 2H), 7.22 (dd, *J* = 2.5, 8.5 Hz, 1H), 7.01 (d, *J* = 2.6 Hz, 1H), 6.93 - 6.87 (m, 1H), 6.86 - 6.81 (m, 1H), 6.76 (s, 1H), 6.62 (d, *J* = 8.6 Hz, 1H), 2.34 (s, 3H); analytical chiral SFC: Rt=1.999 min; ee=97.1%.

Compound (*S*)-17: Dimethyl (*S*)-4'-hydroxy-3'-(6-methyl-2-oxo-3-(1*H*-pyrazol-4-yl)indolin-3-yl)-[1,1'-biphenyl]-,4-dicarboxylate:
A mixture of compound (*S*)-**16** (100 mg, 0.26 mmol), 4,4,5,5-tetramethyl-2-(diethyl 2,4-dicarboxylatephenyl)-1,3-dioxolane (CAS 1186376-95-3, 181 mg, 0.52 mmol), $Pd(dppf)Cl_2$ (19 mg, 0.026 mmol) and Na_2CO_3 (83 mg, 0.78 mmol) in dioxane (3 mL) and water (0.6 mL) was stirred at 90°C for 12 h under an N_2 atmosphere. The RM was diluted with water (50 mL) and extracted with EtOAc (20 mL*3). The combined org. layers were dried over anhydrous Na_2SO_4, filtered and concentrated to give a residue that was purified by FC (PE : EtOAc 0-100%) and prep-HPLC to give the title

compound (70 mg, 51% yield) as a white solid. LC-MS: Rt=0.919 min, m/z = 526.2 $[M+H]^+$.

Compound (*S*)-2: (*S*)-4'-Hydroxy-3'-(6-methyl-2-oxo-3-(1*H*-pyrazol-4-yl)indolin-3-yl)-[1,1'-biphenyl]-2,4-dicarboxylic acid
Aq. NaOH (c = 4 mol/L) was added to a solution of compound (*S*)-**17** (33.5 mg, 0.067 mmol) in MeOH. The RM was stirred overnight, then cooled to RT and ca. 0.3 mL aq. HCl (c = 4.0 mol/L) were added. EtOAc was added and the layers were separated, washed with brine and EtOAc, dried over MgSO4, filtered and concentrated in vacuum. The resulting suspension in EtOAc was filtered and the solid was washed with EtOAc. The solid was dried in HV at 55°C to give the title compound (*S*)**-2** as an off-white solid (23.9 mg, 76% yield): ^{1}H NMR (600 MHz, DMSO-*d*6): δ 13.01 (s, br, 3H), 10.29 (s, 1H), 9.70 (s, 1H), 8.12 (d, *J* = 1.8 Hz, 1H), 8.00 (dd, *J* = 8.0, 1.9 Hz, 1H), 7.41 (d, *J* = 8.0 Hz, 1H), 7.38 (s, 2H), 7.14 (dd, *J* = 8.2, 2.4 Hz, 1H), 6.93 (d, *J* = 7.5 Hz, 1H), 6.87 (d, *J* = 2.3 Hz, 1H), 6.72 – 6.67 (m, 2H), 6.64 (s, 1H), 2.27 (s, 3H); ^{13}C NMR (151 MHz, DMSO-*d*6) δ 179.9, 169.5, 166.7, 154.8, 144.8, 142.4, 136.8, 131.2, 131.1, 130.5, 130.1, 130.0, 129.7, 128.8, 128.0, 123.6, 121.5, 118.5, 115.1, 109.7, 52.8, 21.3 (found 22 of 26 C); HRMS (m/z): $[M-H]^-$ calcd. for $C_{26}H_{19}N_3O_6$, 468.12011; found, 468.12049.

Compound (*R*)-2: (*R*)-4'-Hydroxy-3'-(6-methyl-2-oxo-3-(1*H*-pyrazol-4-yl)indolin-3-yl)-[1,1'-biphenyl]-2,4-dicarboxylic acid was prepared in exact analogy, using (*R*)-**16** instead of (*S*)-**16**: ^{1}H NMR (600 MHz, DMSO-*d*6): δ 12.99 (s, br, 3H), 10.29 (s, 1H), 9.69 (s, 1H), 8.13 (d, *J* = 1.9 Hz, 1H), 8.01 (dd, *J* = 8.0, 1.9 Hz, 1H), 7.42 (d, *J* = 8.0 Hz, 1H), 7.37 (s, 2H), 7.14 (dd, *J* = 8.2, 2.4 Hz, 1H), 6.92 (d, *J* = 7.5 Hz, 1H), 6.86 (d, *J* = 2.3 Hz, 1H), 6.72 – 6.68 (m, 2H), 6.64 (s, 1H), 2.27 (s, 3H). ^{13}C NMR (151 MHz, DMSO-*d*6) δ 179.9, 169.4, 166.6, 154.8, 145.0 142.4, 136.8, 131.2, 130.5, 130.1, 130.0, 129.6, 128.8, 128.0, 123.6, 121.5, 118.5, 115.1, 109.7, 52.7, 21.3 (found 21 of 26 C); HRMS (m/z): $[M-H]^-$ calcd. for $C_{26}H_{19}N_3O_6$, 468.12011; found, 468.12066.

Compounds **3**-**13** were synthesized in analogy to Supplementary Fig. 16 and to published procedures[17,18] by (single or sequential) additions of a substituted phenol and/or an aryl turbo-*Grignard* reagent (obtained from the respective aryl iodide) to a

suitably substituted isatine derivative, followed by e. g. a Suzuki coupling and suitable deprotection steps (ester hydrolysis, demethylation of aromatic OMe group):

Compound 3: (*R*,*S*)-3-Hydroxy-3-(2-hydroxy-5-isopropylphenyl)-6-methylindolin-2-one:
^{1}H NMR (400 MHz, DMSO-*d*6); δ 10.15 (s, 1H), 9.00 (s, 1H), 7.53 (d, *J* = 2.3 Hz, 1H), 6.94 (dd, *J* = 8.1, 2.3 Hz, 1H), 6.68 (d, *J* = 7.4 Hz, 1H), 6.63 – 6.58 (m, 2H), 6.50 (d, *J* = 8.1 Hz, 1H), 6.29 (s, 1H), 2.83 (hept, *J* = 6.9 Hz, 1H), 2.24 (s, 3H), 1.20 (d, *J* = 6.9 Hz, 6H); ^{13}C NMR (151 MHz, DMSO-*d*6) δ 178.8, 151.6, 143.2, 138.0, 137.8, 130.3, 127.5, 125.6, 124.8, 123.6, 121.4, 114.6, 109.7, 75.3, 32.9, 24.4, 24.3, 21.3. HRMS (m/z): $[M-H]^-$ calcd. for $C_{18}H_{19}NO_3$, 296.12922; found 296.12925.

Compound 4: (*R*,*S*)-3-Hydroxy-3-(2-hydroxy-5-isopropylphenyl)-1,6-dimethylindolin-2-one:
^{1}H NMR (400 MHz, DMSO-*d*6) δ 8.97 (s, 1H), 7.58 (d, *J* = 2.3 Hz, 1H), 6.94 (dd, *J* = 8.2, 2.3 Hz, 1H), 6.79 (s, 1H), 6.76 – 6.64 (m, 2H), 6.48 (d, *J* = 8.1 Hz, 1H), 6.35 (s, 1H), 3.11 (s, 3H), 2.90 – 2.80 (m, 1H), 2.30 (s, 3H), 1.21 (d, *J* = 6.9 Hz, 6H); ^{13}C NMR (151 MHz, DMSO-*d*6) δ 177.2, 151.3, 144.6, 138.3, 137.9, 129.5, 127.5, 125.7, 124.6, 123.2, 122.0, 114.6, 108.6, 74.8, 32.9, 25.9, 24.3, 21.4; HRMS (m/z): $[M-H]^-$ calcd. for $C_{19}H_{21}NO_3$, 310.14487; found 310.14464.

Compound 5: (*R*,*S*)-3-(5-Chloro-2-methoxyphenyl)-3-hydroxy-6-(trifluoromethyl)indolin-2-one:
^{1}H NMR (400 MHz, DMSO-*d*6) δ 10.67 (br s, 1H), 7.80 (d, *J* = 2.7 Hz, 1H), 7.36 (dd, *J* = 8.7, 2.7 Hz, 1H), 7.22 – 7.18 (m, 1H), 7.08 – 7.03 (m, 2H), 6.94 (d, *J* = 8.8 Hz, 1H), 6.90 (s, 1H), 3.42 (s, 3H); ^{13}C NMR (151 MHz, DMSO-*d*6) δ 177.3, 154.3, 143.9, 136.6, 131.3, 129.5 (q, *J* = 31.5 Hz), 128.7, 126.8, 124.4, 124.3, 124.1 (*q*, *J* = 272.3 Hz), 118.4 (q, *J* = 4.1 Hz), 113.5, 105.2 (q, *J* = 3.9 Hz), 74.2, 56.0. ^{19}F NMR (376 MHz, DMSO-*d*6): δ -61.1; HRMS (m/z): $[M-H]^-$ calcd. for $C_{16}H_{11}NO_3ClF_3$, 356.03068; found 356.03070.

Compound 6: (*R*,*S*)-3-(5-Chloro-2-hydroxyphenyl)-3-hydroxyindolin-2-one:
^{1}H NMR (400 MHz, DMSO-*d*6) δ 10.28 (br s, 1H), 9.62 (br s, 1H), 7.72 – 7.65 (m, 1H), 7.20 – 7.09 (m, 2H), 6.88 – 6.77 (m, 3H), 6.65 – 6.42 (m, 2H);^{13}C NMR (151

MHz, DMSO-*d*6) δ 177.7, 152.6, 143.2, 132.4, 130.2, 128.8, 127.9, 126.8, 123.7, 122.1, 121.2, 116.5, 109.1, 74.9; HRMS (m/z): $[M-H]^-$ calcd. for $C_{14}H_{10}NO_3Cl$, 274.02765; found 274.02749.

Compound 7: (*R*,*S*)-3-Hydroxy-3-(2-hydroxyphenyl)-6-(trifluoromethyl)indolin-2-one: ^{1}H NMR (400 MHz, DMSO-*d*6) δ 10.53 (br s, 1H), 9.40 (br s, 1H), 7.75 (dd, *J* = 7.7, 1.7 Hz, 1H), 7.22 – 7.16 (m, 1H), 7.12 (m, 1H), 7.04 – 6.96 (m, 2H), 6.92 – 6.85 (m, 1H), 6.73 – 6.56 (m, 2H); ^{19}F NMR (376 MHz, DMSO-*d*6) δ -61.0; ^{13}C NMR (151 MHz, DMSO-*d*6) δ 178.0, 153.4, 144.0, 137.6, 129.1 (q, *J* = 31.4 Hz), 128.6, 127.1, 127.0, 124.3, 124.2 (q, *J* = 272.2 Hz), 118.5, 118.2 (q, *J* = 4.1 Hz), 114.8, 105.0 (q, *J* = 4.0 Hz), 74.7. HRMS (m/z): $[M-H]^-$ calcd. for $C_{15}H_{10}NO_3F_3$, 308.05400; found 308.05385.

Compound 8: (*R*,*S*)-3-(5-Chloro-2-hydroxyphenyl)-3-hydroxy-6-methylindolin-2-one ^{1}H NMR (600 MHz, DMSO-*d*6): δ 10.24 (s, br, 1H), 9.61 (s, br, 1H), 7.66 (d, *J* = 2.7 Hz, 1H), 7.12 (dd, *J* = 8.5, 2.8 Hz, 1H), 6.71 (d, *J* = 7.4 Hz, 1H), 6.64 – 6.58 (m, 3H), 6.48 (s, br, 1H), 2.25 (s, 3H); ^{13}C NMR (151 MHz, DMSO-*d*6) δ 178.0, 152.6, 143.3, 138.4, 130.4, 129.6, 127.8, 126.8, 123.5, 122.0, 121.6, 116.5, 109.9, 74.8, 21.3, HRMS (m/z): $[M-H]^-$ calcd. for $C_{15}H_{12}NO_3Cl$, 288.04330; found, 288.04348.

Compound 9: (*R*,*S*)-6-Chloro-3-(5-chloro-2-hydroxyphenyl)-3-hydroxyindolin-2-one: ^{1}H NMR (600 MHz, DMSO-*d*6): δ 10.48 (s, 1H), 9.71 (s, br, 1H), 7.69 (d, *J* = 2.7 Hz, 1H), 7.15 (dd, *J* = 8.5, 2.8 Hz, 1H), 6.87 – 6.83 (m, 2H), 6.81 (d, *J* = 1.7 Hz, 1H), 6.68 (s, br, 1H), 6.61 (d, *J* = 8.5 Hz, 1H); ^{13}C NMR (151 MHz, DMSO-*d*6) δ 177.6, 152.5, 144.8, 133.0, 131.4, 129.6, 128.2, 126.8, 125.1, 122.2, 120.9, 116.5, 109.2, 74.3; HRMS (m/z): $[M-H]^-$ calcd. for $C_{14}H_9NO_3Cl_2$, 307.98867; found, 307.98897.

Compound 10: (*R*,*S*)-4'-Hydroxy-3'-(3-hydroxy-6-methyl-2-oxoindolin-3-yl)-[1,1'-biphenyl]-4-carboxylic acid:^{1}H NMR (400 MHz, DMSO-*d*6) δ 12.87 (s, 1H), 10.20 (s, 1H), 9.61 (s, 1H), 8.13 – 8.06 (m, 1H), 8.01 (d, *J* = 8.1 Hz, 2H), 7.76 (d, *J* = 8.1 Hz, 2H), 7.50 (d, *J* = 7.6 Hz, 1H), 6.74 (dd, *J* = 14.9, 7.9 Hz, 2H), 6.62 (s, 2H), 6.44 (s, 1H), 2.26 (s, 3H);^{13}C NMR (101 MHz, DMSO-*d*6) δ 179.0, 167.8, 154.7, 145.2, 143.8, 138.7, 130.5, 130.4, 129.5, 129.3, 129.1, 127.3, 126.4, 126.2, 124.1, 122.0,

116.0, 115.0, 110.3, 75.6, 21.8 (21 of 22 Cs found); HRMS (m/z): $[M-H]^-$ calcd. for $C_{22}H_{17}NO_5$, 374.10340; found, 374.10270.

Compound 11: (*R*,*S*)-3'-(6-chloro-3-hydroxy-2-oxoindolin-3-yl)-4'-hydroxy-[1,1'-biphenyl]-2-carboxylic acid:^{1}H NMR (600 MHz, DMSO-*d*6): δ 12.80 (s, br, 1H), 10.42 (s, 1H), 9.52 (s, br, 1H), 7.74 (d, *J* = 2.4 Hz, 1H), 7.67 (dd, *J* = 7.7, 1.4 Hz, 1H), 7.57 – 7.52 (m, 1H), 7.44 – 7.38 (m, 2H), 7.11 (dd, *J* = 8.2, 2.4 Hz, 1H), 6.90 – 6.85 (m, 2H), 6.81 (d, *J* = 1.7 Hz, 1H), 6.64 (d, *J* = 8.2 Hz, 1H), 6.51 (s, br, 1H). ^{13}C NMR (151 MHz, DMSO-*d*6) δ 178.3, 170.3, 152.9, 144.7, 141.0, 132.8, 131.9, 130.9, 130.6, 130.3, 129.0, 128.3, 127.3, 127.1, 126.5, 125.4, 120.7, 114.7, 109.1, 74.6. HRMS (m/z): $[M-H]^-$ calcd. for $C_{21}H_{14}NO_5Cl$, 394.04878; found, 394.04846.

Compound 12: (*R*,*S*)-3-(2-Hydroxy-5-methylphenyl)-3-(1*H*-pyrazol-4-yl)-6-(trifluoromethyl)indolin-2-one:
^{1}H NMR (600 MHz, DMSO-*d*6): δ 12.93 (s, br, 1H), 10.63 (s, br, 1H), 9.29 (s, br, 1H), 7.44 (s, br, 2H), 7.26 – 7.20 (m, 2H), 7.03 (d, *J* = 1.7 Hz, 1H), 6.89 (dd, *J* = 8.2, 2.2 Hz, 1H), 6.63 (d, *J* = 2.2 Hz, 1H), 6.52 (d, *J* = 8.0 Hz, 1H), 2.12 (s, 3H); ^{13}C NMR (151 MHz, DMSO-*d*6) δ 179.6, 152.3, 143.2, 139.0, 130.0, 129.0, 127.9 (q, *J* = 27.0 Hz), 127.8, 126.8, 124.3 (q, *J* = 272.1 Hz), 124.2, 123.4, 121.6, 118.1 (q, *J* = 4.1 Hz), 117.2, 114.9, 104.9 (q, *J* = 3.9 Hz), 55.0, 20.4; ^{19}F NMR (565 MHz, DMSO-*d*6) δ -60.8; HRMS (m/z): $[M-H]^-$ calcd. for $C_{19}H_{14}N_3O_2F_3$, 372.09654; found, 372.09655.

Compound 13: (*R*,*S*)-3-Hydroxy-3-(2-hydroxy-5-methylphenyl)-6-(trifluoromethyl)indolin-2-one:
^{1}H NMR (600 MHz, DMSO-*d*6): δ 10.53 (s, br, 1H), 9.13 (s, br, 1H), 7.56 (d, *J* = 2.3 Hz, 1H), 7.19 – 7.16 (m, 1H), 7.02 – 7.00 (m, 2H), 6.93 – 6.90 (m, 1H), 6.60 (s, br, 1H), 6.49 (d, *J* = 8.0 Hz, 1H), 2.27 (s, 3H); ^{13}C NMR (151 MHz, DMSO-*d*6) δ 178.0, 151.1, 144.0, 137.8, 129.0 (q, J =,31.3 Hz), 128.9, 127.4, 126.8, 126.7, 124.3, 124.2 (q, *J* = 272.2 Hz), 118.2, (q, *J* = 4.5 Hz), 114.7, 105.0 (q, J = 4.0 Hz), 74.7, 20.5; ^{19}F NMR (565 MHz, DMSO-*d*6) δ -60.9; HRMS (m/z): $[M-H]^-$ calcd. for $C_{16}H_{12}NO_3F_3$, 322.06965; found, 322.06981.

Reporter cell system for NanoBiT assay

CHO IL-36R or CHO IL-1R1 reporter cell lines, prepared internally (Klemens Kaupmann), based on tagged IL-1RAcP and Myd88 proteins using the NanoBiT complementation system, were seeded at a density of 10'000 cells/well in 30 μL of OptiMEM + 0.1 % FCS medium using 384-well plates (white, TCT), for 24 hours. Before the treatment, the medium was removed, 20 μL of NanoGlo substrate, (#N2012, Promega) were added to the wells. Following substrate addition, 240 nL of compound were added to the wells using an ECHO dispenser device. Then, 3.76 μL of the ligand IL-36γ (Novartis) at 200 ng/mL end concentration or recombinant IL-1β (Novartis) at 500 pg/mL end concentration were added to the mix. Luminescence was measured at 405 nm after 30 min incubation (Pherastar reader).

Supplementary References

1. Evans, P. R. An introduction to data reduction: space-group determination, scaling and intensity statistics. *Acta Crystallogr. D Biol. Crystallogr.* **67**, 282–292 (2011).
2. Brünger, A. T. Free R value: a novel statistical quantity for assessing the accuracy of crystal structures. *Nature* **355**, 472–475 (1992).
3. Karplus, P. A. & Diederichs, K. Linking crystallographic model and data quality. *Science* **336**, 1030–1033 (2012).
4. Karplus, P. A. & Diederichs, K. Assessing and maximizing data quality in macromolecular crystallography. *Curr. Opin. Struct. Biol.* **34**, 60–68 (2015).
5. Chen, V. B. *et al.* MolProbity: all-atom structure validation for macromolecular crystallography. *Acta Crystallogr. D Biol. Crystallogr.* **66**, 12–21 (2010).
6. Lingel, A. *et al.* Comprehensive and high-throughput exploration of chemical space using broadband ^{19}F NMR-based screening. *Angew. Chem. Int. Ed. Engl.* **59**, 14809–14817 (2020).
7. Feeney, J., Batchelor, J. G., Albrand, J. P. & Roberts, G. C. K. The effects of intermediate exchange processes on the estimation of equilibrium constants by NMR. *Journal of Magnetic Resonance (1969)* **33**, 519–529 (1979).
8. Dalvit, C. *et al.* Sensitivity improvement in ^{19}F NMR-based screening experiments: theoretical considerations and experimental applications. *J. Am. Chem. Soc.* **127**, 13380–13385 (2005).

9. Liebschner, D. A. *et al.* Polder maps: improving OMIT maps by excluding bulk solvent. *Acta Crystallogr. D Biol. Crystallogr.* **73**, 148–157 (2017).

10. Skinner, S. P. *et al.* CcpNmr AnalysisAssign: a flexible platform for integrated NMR analysis. *J. Biomol. NMR* **66**, 111–124 (2016).
Thomas, C., Bazan, J. F. & Garcia, K. C. Structure of the activating IL-1 receptor signaling complex. *Nat. Struct. Mol. Biol.* **19**, 455–457 (2012).

11. Thomas, C., Bazan, J. F. & Garcia, K. C. Structure of the activating IL-1 receptor signaling complex. *Nat. Struct. Mol. Biol.* **19**, 455–457 (2012).

12. Todorovic, V. *et al*. Small molecule IL-36 gamma antagonist as a novel therapeutic approach for plaque psoriasis. *Sci. Rep.* **9**, 9089 (2019).

13. Chang, H.-K., Mohan, S.K., & Chin, Y. 1H, 13C and 15N backbone and side chain resonance assignments of human interleukin 1alpha. *Biomol. NMR Assign.* **4**, 59-60 (2010).

14. Krissinel, E. Enhanced Fold Recognition using Efficient Short Fragment Clustering. *J. Mol. Biochem.* **1**, 76–85 (2012).

15. Winn, M. D. *et al.* Overview of the CCP4 suite and current developments. *Acta Crystallogr. D Biol. Crystallogr.* **67**, 235–242 (2011).

16. Priestle, J. P., Schar, H. P., & Grutter, M. G. Crystallographic refinement of interleukin 1 beta at 2.0 A resolution. *Proc. Natl. Acad. Sci. U. S. A*. **86**, 9667-9671 (1989).

17. Hewawasam, P. *et al.* Synthesis and structure-activity relationships of 3-aryloxindoles: a new class of calcium-dependent, large conductance potassium (maxi-K) channel openers with neuroprotective properties. *J. Med. Chem.* **45**, 1487–1499 (2002).

18. Kinthada, L. K. *et al.* Friedel-Crafts alkylations of electron-rich aromatics with 3-hydroxy-2-oxindoles: scope and limitations. *Org. Biomol. Chem.* **12**, 8152–8173 (2014).
