# Supplementary material for: Discovery of a selective and biologically active low-molecular weight antagonist of human interleukin-1β
Source: Nat Commun. 2023 Sep 7;14:5497. doi: 10.1038/s41467-023-41190-0 (PMC10484922; doi:10.1038/s41467-023-41190-0)
Supplement: Supplementary file 3 — Description of Additional Supplementary Files [file 41467_2023_41190_MOESM3_ESM.pdf]

Description of Additional Supplementary Files

Supplementary Software: FRPipe.py is the in-house python script to analyse 19F reporter assay data. It reads preprocessed Bruker spectra and parses accompanying files, enriched with meta data needed for further processing. With this information it groups relevant peak information of individual experiments to determine Ki values of the given small molecule.

The source code is available here: https://github.com/Novartis/FRPipe
