# Supplementary material for: Discovery of a selective and biologically active low-molecular weight antagonist of human interleukin-1β
Source: Nat Commun. 2023 Sep 7;14:5497. doi: 10.1038/s41467-023-41190-0 (PMC10484922; doi:10.1038/s41467-023-41190-0)
Supplement: Supplementary file 5 — Reporting Summary [file 41467_2023_41190_MOESM5_ESM.pdf]

nature portfolio

Corresponding author(s): Ulrich Hommel, Konstanze Hurth, Frederic Bornancin

Last updated by author(s): May 1, 2023

Reporting Summary

Nature Portfolio wishes to improve the reproducibility of the work that we publish. This form provides structure for consistency and transparency in reporting. For further information on Nature Portfolio policies, see our Editorial Policies and the Editorial Policy Checklist.

Statistics

For all statistical analyses, confirm that the following items are present in the figure legend, table legend, main text, or Methods section.

n/a	Confirmed	
☐	☒	The exact sample size (*n*) for each experimental group/condition, given as a discrete number and unit of measurement
☐	☒	A statement on whether measurements were taken from distinct samples or whether the same sample was measured repeatedly
☒	☐	The statistical test(s) used AND whether they are one- or two-sided<br>*Only common tests should be described solely by name; describe more complex techniques in the Methods section.*
☒	☐	A description of all covariates tested
☒	☐	A description of any assumptions or corrections, such as tests of normality and adjustment for multiple comparisons
☐	☒	A full description of the statistical parameters including central tendency (e.g. means) or other basic estimates (e.g. regression coefficient) AND variation (e.g. standard deviation) or associated estimates of uncertainty (e.g. confidence intervals)
☒	☐	For null hypothesis testing, the test statistic (e.g. *F*, *t*, *r*) with confidence intervals, effect sizes, degrees of freedom and *P* value noted<br>*Give P values as exact values whenever suitable.*
☒	☐	For Bayesian analysis, information on the choice of priors and Markov chain Monte Carlo settings
☒	☐	For hierarchical and complex designs, identification of the appropriate level for tests and full reporting of outcomes
☒	☐	Estimates of effect sizes (e.g. Cohen's *d*, Pearson's *r*), indicating how they were calculated

Our web collection on statistics for biologists contains articles on many of the points above.

Software and code

Policy information about availability of computer code

Data collection	DA+ data acquisition software implemented at the SLS beamline PX-II<br>Bruker TopSpin 3.2
Data analysis	PRISM 7.0<br>Sierra Analyser<br>AUTOPROC1.1.7 (20220203)<br>autoBUSTER2.11.8 (20220203)<br>PHASER 2.8.3<br>PHENIX 1.20_4459<br>COOT 0.9.6 EL<br>CCPNMR AnalysisAssign 3.1.0<br>AnalysisScreen 3.0.4<br>nmrglue 0.9<br>peakipy: https://github.com/j-brady/peakipy<br>ChemEx: https://github.com/gbouvignies/ChemEx<br>FRPipe: https://github.com/Novartis/FRPipe

For manuscripts utilizing custom algorithms or software that are central to the research but not yet described in published literature, software must be made available to editors and reviewers. We strongly encourage code deposition in a community repository (e.g. GitHub). See the Nature Portfolio guidelines for submitting code & software for further information.

Data

Policy information about availability of data

All manuscripts must include a data availability statement. This statement should provide the following information, where applicable:

- Accession codes, unique identifiers, or web links for publicly available datasets
- A description of any restrictions on data availability
- For clinical datasets or third party data, please ensure that the statement adheres to our policy

Diffraction data and crystallographic coordinates of the complex between hIL-1b and compound (S)-2 generated in this study have been deposited in the PDB database under accession code 8C3U [http://doi.org/10.2210/pdb8C3U/pdb]. Previously published crystallographic structures used in this study are available from the PDB under accession codes 4DEP [http://doi.org/10.2210/pdb4DEP/pdb] (human IL-1b complex with IL-1R1 and IL-1RAcP), 2I1B [http://doi.org/10.2210/pdb2I1B/pdb] (unliganded human IL-1b), 2KKI [http://doi.org/10.2210/pdb2KKI/pdb] (NMR structure of human IL-1a) and 6P9E [http://doi.org/10.2210/pdb6P9E/pdb] (human IL-36g in complex with A-552). The NMR chemical shift assignments generated in this study have been deposited in the BMRB database under accession codes 51919 [https://dx.doi.org/10.13018/BMR51919] (hIL-1b at 296 K), 51859 [https://dx.doi.org/10.13018/BMR51859] (hIL-1b at 309 K), 51938 [https://dx.doi.org/10.13018/BMR51938] (hIL-1b in complex with (S)-2) and 51950 [https://dx.doi.org/10.13018/BMR59150] (hIL-1bV47A at 296 K and 309 K). Source data are provided with this paper.

Human research participants

Policy information about studies involving human research participants and Sex and Gender in Research.

Reporting on sex and gender	not applicable
Population characteristics	not applicable
Recruitment	not applicable
Ethics oversight	not applicable

Note that full information on the approval of the study protocol must also be provided in the manuscript.

Field-specific reporting

Please select the one below that is the best fit for your research. If you are not sure, read the appropriate sections before making your selection.

☒ Life sciences ☐ Behavioural & social sciences ☐ Ecological, evolutionary & environmental sciences

For a reference copy of the document with all sections, see nature.com/documents/nr-reporting-summary-flat.pdf

Life sciences study design

All studies must disclose on these points even when the disclosure is negative.

Sample size	n>=3 for key data points
Data exclusions	No data exclusion
Replication	All attempts at replication were successful
Randomization	Not applicable
Blinding	Not applicable

Reporting for specific materials, systems and methods

We require information from authors about some types of materials, experimental systems and methods used in many studies. Here, indicate whether each material, system or method listed is relevant to your study. If you are not sure if a list item applies to your research, read the appropriate section before selecting a response.

Materials & experimental systems

n/a	Involved in the study
☐	☒ Antibodies
☐	☒ Eukaryotic cell lines
☒	☐ Palaeontology and archaeology
☒	☐ Animals and other organisms
☒	☐ Clinical data
☒	☐ Dual use research of concern

Methods

n/a	Involved in the study
☒	☐ ChIP-seq
☒	☐ Flow cytometry
☒	☐ MRI-based neuroimaging

Antibodies

Antibodies used	Canakinumab (Ilaris®)
Validation	validation of canakinumab: Figure 3 and reference 10

Eukaryotic cell lines

Policy information about cell lines and Sex and Gender in Research

Cell line source(s)	HEK-Blue™ IL-1b cells that respond specifically to IL-1 (# hkb-il1b, InvivoGen)<br>Normal human dermal fibroblasts (NHDF) from CloneticsTM (LONZA, Cat # CC-2509 (NHDF-Neo), Lot # 0000234242
Authentication	None of the cell lines used were authenticated
Mycoplasma contamination	Cell lines were not tested for mycoplasma contamination
Commonly misidentified lines (See ICLAC register)	There was no misidentified cell line used in the study
